# Inner Hair Cell Loss Disrupts Hearing and Cochlear Function Leading to Sensory Deprivation and Enhanced Central Auditory Gain

**DOI:** 10.3389/fnins.2016.00621

**Published:** 2017-01-18

**Authors:** Richard Salvi, Wei Sun, Dalian Ding, Guang-Di Chen, Edward Lobarinas, Jian Wang, Kelly Radziwon, Benjamin D. Auerbach

**Affiliations:** ^1^Center for Hearing and Deafness, University at BuffaloBuffalo, NY, USA; ^2^Callier Center, University of Texas at DallasDallas, TX, USA; ^3^School of Human Communication Disorders, Dalhousie UniversityHalifax, NS, Canada

**Keywords:** inner hair cells, carboplatin, central auditory system, auditory gain, auditory cortex, tinnitus, hyperacusis

## Abstract

There are three times as many outer hair cells (OHC) as inner hair cells (IHC), yet IHC transmit virtually all acoustic information to the brain as they synapse with 90–95% of type I auditory nerve fibers. Here we review a comprehensive series of experiments aimed at determining how loss of the IHC/type I system affects hearing by selectively destroying these cells in chinchillas using the ototoxic anti-cancer agent carboplatin. Eliminating IHC/type I neurons has no effect on distortion product otoacoustic emission or the cochlear microphonic potential generated by OHC; however, it greatly reduces the summating potential produced by IHC and the compound action potential (CAP) generated by type I neurons. Remarkably, responses from remaining auditory nerve fibers maintain sharp tuning and low thresholds despite innervating regions of the cochlea with ~80% IHC loss. Moreover, chinchillas with large IHC lesions have surprisingly normal thresholds in quiet until IHC losses exceeded 80%, suggesting that only a few IHC are needed to detect sounds in quiet. However, behavioral thresholds in broadband noise are elevated significantly and tone-in-narrow band noise masking patterns exhibit greater remote masking. These results suggest the auditory system is able to compensate for considerable loss of IHC/type I neurons in quiet but not in difficult listening conditions. How does the auditory brain deal with the drastic loss of cochlear input? Recordings from the inferior colliculus found a relatively small decline in sound-evoked activity despite a large decrease in CAP amplitude after IHC lesion. Paradoxically, sound-evoked responses are generally larger than normal in the auditory cortex, indicative of increased central gain. This gain enhancement in the auditory cortex is associated with decreased GABA-mediated inhibition. These results suggest that when the neural output of the cochlea is reduced, the central auditory system compensates by turning up its gain so that weak signals once again become comfortably loud. While this gain enhancement is able to restore normal hearing under quiet conditions, it may not adequately compensate for peripheral dysfunction in more complex sound environments. In addition, excessive gain increases may convert recruitment into the debilitating condition known as hyperacusis.

## Sensorineural hearing loss and the audiogram

The audiogram is often considered the gold standard for assessing sensorineural hearing loss (HL). Individuals with pure tone thresholds of ≤20 dB HL would be classified as having normal hearing. However, there is growing awareness that the pure tone audiogram fails to detect certain forms of cochlear pathology and auditory processing deficits. This has led to the concept of “hidden hearing loss,” i.e., the realization that significant auditory perceptual deficits can exist in listeners with normal hearing thresholds, a condition that can exist when there is considerable IHC and/or auditory nerve fiber degeneration (Schaette and McAlpine, 2011; Plack et al., 2014; Lobarinas et al., 2016). Hidden hearing loss is likely involved in some cases of auditory neuropathy and central auditory processing disorders, which are characterized by temporal processing deficits, impaired speech perception, and difficulties hearing in noisy environments (Kraus et al., 2000; Zeng et al., 2005). It may also contribute to other auditory perceptual disorders such as tinnitus and hyperacusis (Schaette and McAlpine, 2011; Hickox and Liberman, 2014). It is therefore imperative to develop ways for clinically assessing hidden hearing loss and determining the consequences of IHC/auditory nerve damage on peripheral and central auditory processing.

Electrocochleography (ECochG) can be used to interrogate the functional status of different structures in the cochlea and identify “hidden” damage to inner hair cells (IHC), outer hair cells (OHC), the IHC/type I auditory nerve fiber synapse, and spiral ganglion neurons (SGN). Sensorineural hearing loss is a complex phenomenon that not only involves the cochlea, but also numerous structures in the central auditory system capable of partially compensating for these cochlear deficits. Therefore, a more complete understanding of sensorineural hearing loss not only requires assessment with ECochG, but also examination of the neurophysiological changes occurring in the central auditory pathway. In this review, we will discuss our results from a comprehensive series of electrophysiological, neuroanatomical, behavioral, and neuropharmacological experiments in a chinchilla animal model of carboplatin-induced ototoxicity in which there is selective damage to the IHC and type I auditory nerve fibers that exclusively innervate the IHC. These studies illustrate how ECochG can be used to identify damage to the IHC and type I neurons that goes undetected (i.e., hidden) by the pure tone audiogram. Electrophysiological recordings from the inferior colliculus (IC) and auditory cortex (ACx) reveal how weak neural signals from a damaged cochlea are amplified as they ascend through the central auditory pathway. Finally, we discuss a few simple psychophysical tests we have shown can identify hearing deficits associated with damage to IHC and type I neurons.

## Carboplatin-induced IHC and type I lesions

**Figure 1:**
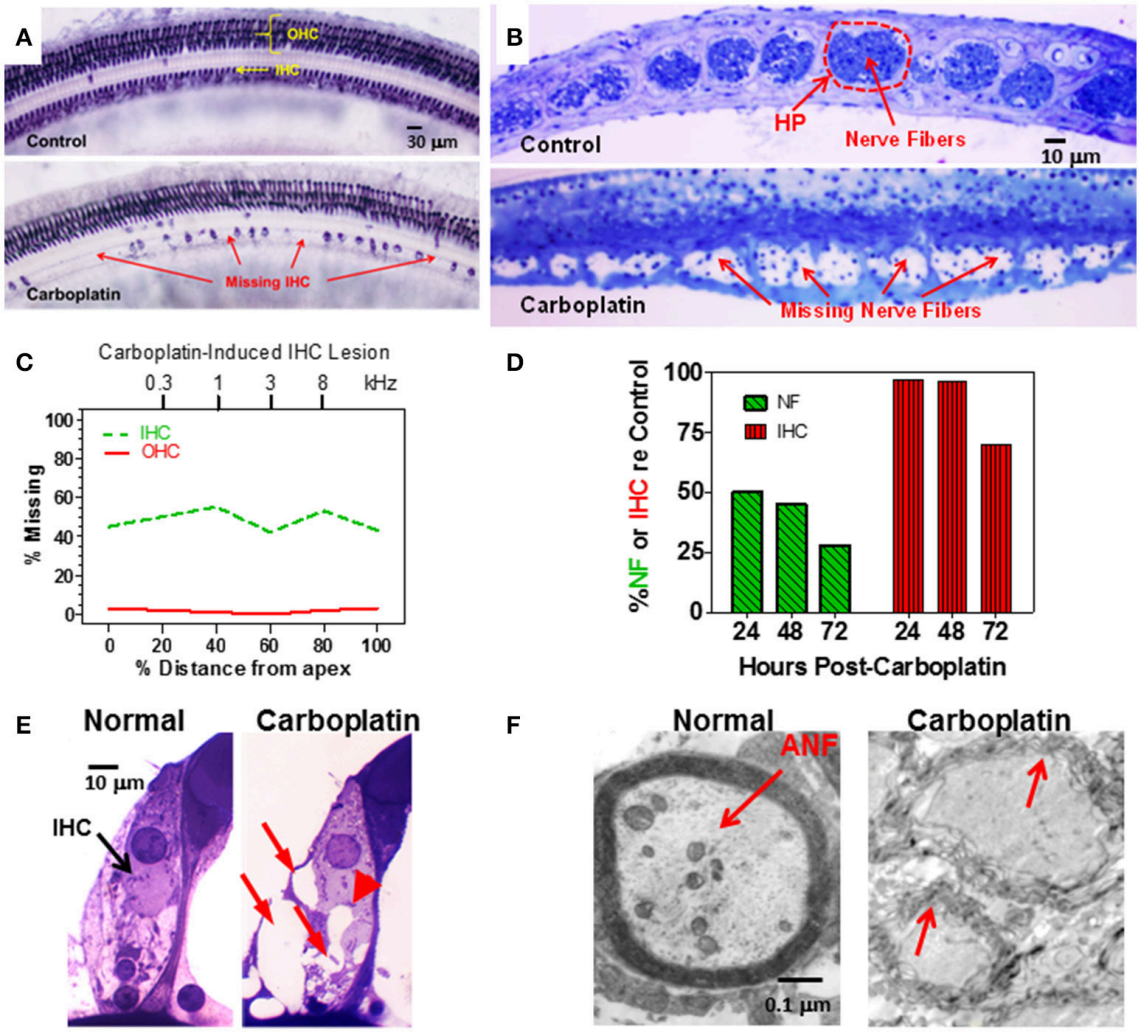
**(A)** Photomicrographs of a surface preparation of the organ of Corti stained with succinate dehydrogenase, a metabolic enzyme highly expressed in OHC and IHC, but not supporting cells. Control (upper panel) shows strong staining of all OHC and IHC. One month after a moderate dose of carboplatin (50–75 mg/kg, i.p.) there are patches of stained IHC separated by large regions of missing IHC. OHC were present and appeared normal. **(B)** Photomicrographs of thin sections stained with toluidine blue taken tangential to the habenula perforata. Dashed line (upper panel) showing the darkly stained nerve fibers in the openings in the habenular perforata (HP) in the osseous spiral lamina (dashed line) of a normal control ear. Each habenular opening in control ears is filled with nerve fibers (upper panel) whereas in carboplatin-treated ears (bottom panel), many nerve fibers are missing in the habenular openings. **(C)** Schematic of a cochleogram showing the typical pattern of IHC loss induced by a moderate dose of carboplatin. In this depiction, roughly 40–50% of the IHC were missing along the length of the cochlea whereas OHC were intact. The cochleogram shows the percentage of missing IHC and OHC as a function of percent distance from the apex of the cochlea; cochlear position related to frequency on the upper x-axis. **(D)** Carboplatin induced a large and rapid loss of nerve fibers (NF) in the habenula perforata 24–72 h post-treatment. Significant nerve fiber (NF) loss occurred 24 h post-treatment; IHC occurred several days later. **(E)** Photomicrographs illustrating the condition of the synaptic region at the base of the IHC of a normal control (left) and a carboplatin-treated animal (right). At 24 h post-treatment, many large vacuoles (red arrows) were observed at the afferent terminals of the carboplatin-treated chinchilla unlike the control. Swelling distorted the basal pole of the IHC in carboplatin treated (arrowhead) animal. **(F)** Transmission electron micrograph show thick myelin sheath around a normal auditory nerve fiber (ANF). Carboplatin caused significant demyelination 24–72 h post-treatment (red arrows). Data schematized from Hofstetter et al. (1997b), Ding et al. (1999, 2001), and Wang et al. (2003).

Cisplatin and other platinum based anti-cancer drugs are generally more toxic to OHC than IHC, with hair cell lesions generally progressing from the base toward the apex as the dose and duration of treatment increases (Boettcher et al., 1992; Rybak et al., 2007). Carboplatin is a second generation antineoplastic agent that is considered much less ototoxic than cisplatin (Ettinger et al., 1994), a view consistent with most studies in animal models (Saito et al., 1989; Ding et al., 1999). However, when low-to-moderate doses of carboplatin (50–75 mg/kg, i.p.) were systemically administered to chinchillas, it induced an unusual lesion that preferentially damaged IHC (Figure 1A), type I auditory nerve fibers (Figure 1B) and SGN. OHC damage was only observed at extremely high doses of carboplatin (200 mg/kg, i.p.) (Takeno et al., 1994, 1998; Hofstetter et al., 1997a; Wang et al., 1997; Ding et al., 1999). Unlike other ototoxic drugs, the IHC lesion was characterized by a relatively uniform loss of hair cells along the entire length of the cochlea (Trautwein et al., 1996; Hofstetter et al., 1997a; Figure 1C). Due to the systemic nature of treatment, hall cell lesions were similar in both ears (Hofstetter, 1996; Hofstetter et al., 1997a).

To gain insights into the time course of carboplatin-mediated damage, we counted the number of IHC and nerve fibers in the habenula perforata 24–72 h after treatment with a moderate dose of carboplatin (50 mg/kg, i.p.) (Wang et al., 2003). Surprisingly, 24 h after carboplatin treatment, only 50% of the nerve fibers in the habenula perforata were present whereas there was no loss of IHC (Figure 1D). Significant IHC loss was first observed 3 days post-carboplatin, but by this time only ~25% of the nerve fibers were still present. These results suggest that the auditory nerve fibers and their afferent synapses are especially susceptible to carboplatin ototoxicity. To explore this possibility, transmission electron microscopy was used to examine the morphological condition of the type I afferent synapse at the base of the IHC. At 24 h post-carboplatin, numerous vacuoles were present around afferent terminals at the base of the IHC (Figure 1E; Ding et al., 1997, 1998). Damage to the afferent nerve terminals, IHC and SGN increased considerably between 24 and 72 h whereas the morphology of the OHC remained remarkably normal. Vacuoles were also present on the proximal nerve fibers and transmission electron microscopy revealed significant loss of myelin around the nerve fibers 24–72 h post-treatment (Figure 1F; Ding et al., 2002; Wang et al., 2003). Taken together, these results indicate that moderate doses of carboplatin can selectively damage IHC and type I afferent neurons.

## Massive IHC lesions have little effect on distortion product otoacoustic emissions

**Figure 2:**
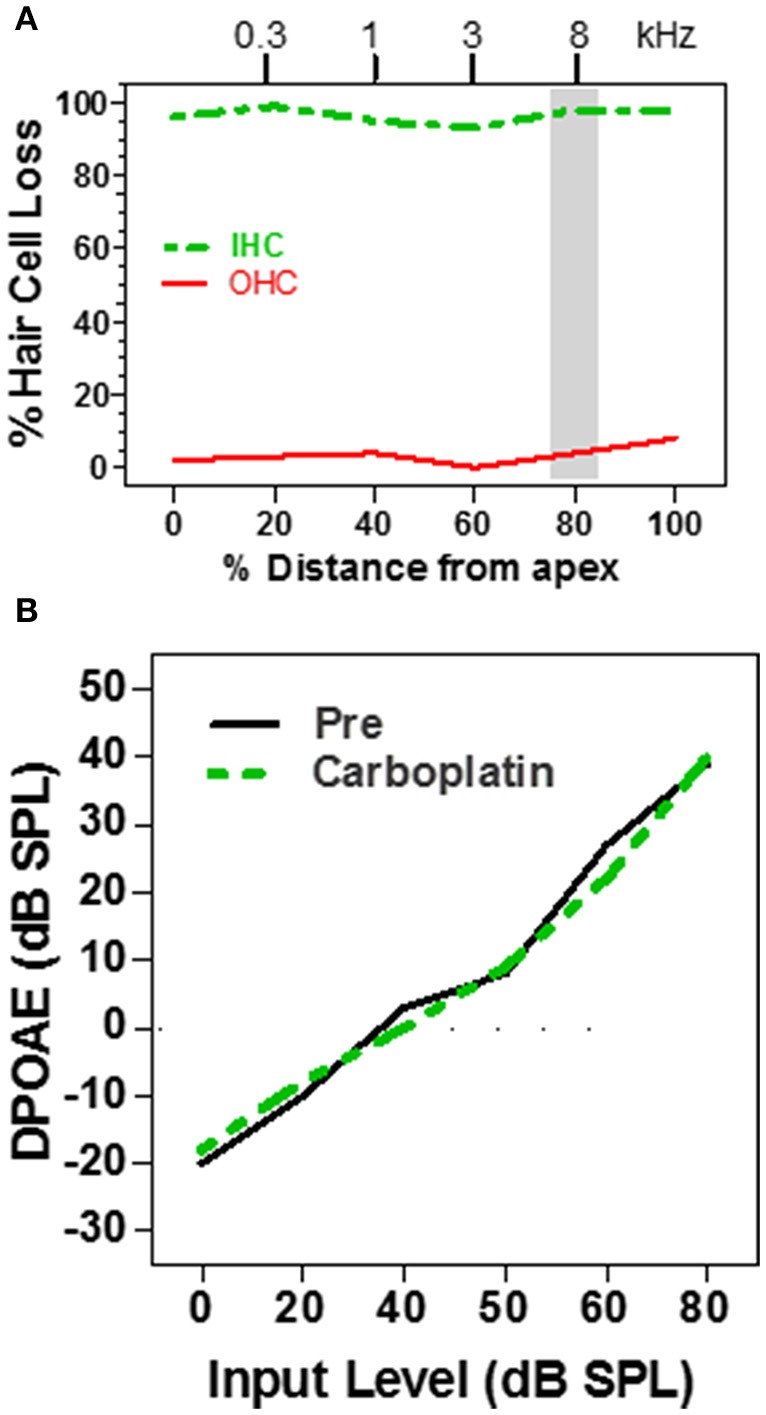
**(A)** Schematic cochleogram showing a very large IHC lesion with little OHC loss induced by a high dose of carboplatin. IHC lesion extends over nearly the entire length of the cochlea. **(B)** Schematic DPOAE input/output function before and after carboplatin treatment at f1 and f2 frequencies corresponding to the shaded region in the cochleogram in **(A)**. DPOAE input/output function was normal several months post-carboplatin despite massive IHC loss, but retention of most OHC. Data schematized from Hofstetter et al. (1997a).

Distortion product otoacoustic emissions (DPOAE), which depend on OHC somatic electromotility (Brownell, 1990; Liberman et al., 2002), provide a noninvasive method for assessing the functional integrity of the cochlea (Brown et al., 1989; Schrott et al., 1991; Hofstetter et al., 1997a) and are widely used to screen for cochlear hearing loss in infants and adults (Stanton et al., 2005; Jakubíková et al., 2009). Subjects with normal DPOAE pass the screening test and are generally believed to have normal hearing; however, since DPOAE are specifically sensitive to OHC function, this is not always correct, for example in patients with auditory neuropathy (Abdala et al., 2000). Since moderate doses of carboplatin selectively damage the IHC while ostensibly leaving the OHC intact, DPOAE might be expected to be normal in ears with just IHC loss. To test this hypothesis, DPOAE input/output functions were measured in chinchillas before and after treatment with a moderate to high dose of carboplatin (Trautwein et al., 1996; Wake et al., 1996a; Hofstetter et al., 1997a). In some animals, carboplatin treatment caused near complete loss of IHC along the entire length of the cochlea, but failed to damage the OHC (Figure 2A). In such cases, where nearly all the IHC were missing but the OHC were intact, the DPOAE input/output functions were completely normal (Figure 2B; Hofstetter et al., 1997a). Thus, the presence of normal DPOAE does not mean that the cochlea is structurally intact.

## Moderate IHC lesion has little effect on threshold

**Figure 3:**
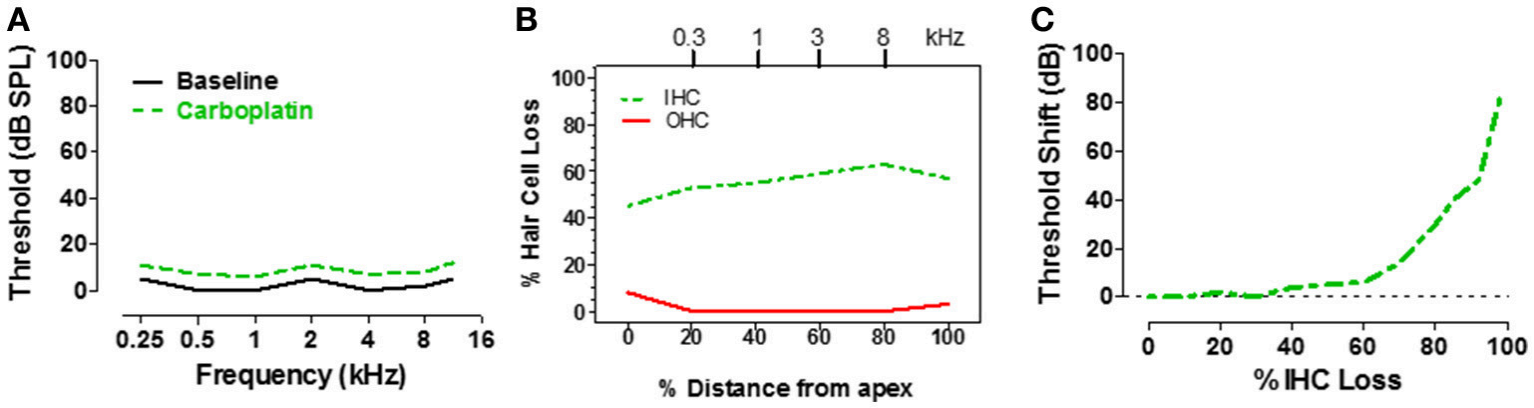
**(A)** Schematic of pure tone audiogram obtained pre- and post-carboplatin in a chinchilla with ~50–60% IHC and an intact OHC population **(B)**. The post-carboplatin thresholds (green) were slightly increased from baseline (black). **(B)** Schematic of cochleogram showing 50–60% IHC and minimal OHC loss following carboplatin treatment (audiometric profile for such lesions depicted in **A**). Percent distance from the apex of cochlea shown on x-axis; position in the cochlea related to frequency on upper x-axis. **(C)** Schematic showing the approximate relationship between the threshold shift vs. the percent IHC loss induced by carboplatin. Thresholds remained nearly normal up to about 60% IHC loss, but then increased steeply once the IHC lesion exceeds 80%. Data schematized from Lobarinas et al. (2013).

IHC make one-to-one synaptic contact with the type I auditory nerve fibers, providing the only pathway through which acoustic information is relayed to the central auditory system (Spoendlin and Baumgartner, 1977). Therefore, massive loss of IHC would greatly reduce input to the central auditory system and should drastically disrupt hearing. To determine how IHC loss affects hearing thresholds, pure tone audiograms were measured in chinchillas using an avoidance conditioning paradigm before and 1–2 months after moderate to high doses of carboplatin designed to induce a range of IHC lesions (Salvi et al., 1978; Lobarinas et al., 2013). After completing the hearing tests, the cochleae were harvested to determine the magnitude and type of cochlear lesion. The schematic audiogram (Figure 3A) and schematic cochleogram (Figure 3B) illustrate the results obtained when carboplatin induced a moderate IHC lesion, but no OHC damage. In these cases, thresholds in quiet were surprisingly unaffected, increasing very little despite the fact that 40–60% of the IHC were missing (Lobarinas et al., 2013). To understand the relationship between hearing loss and IHC loss, the threshold shifts post-carboplatin were plotted as a function of percent IHC loss as schematized in Figure 3C. Hearing thresholds were largely unaffected by small IHC lesions (<35%). Threshold shifts gradually increased with moderate IHC lesions (40–75%), but then increased substantially once the IHC lesions exceeded 80%. One interpretation of these results is that the pure tone audiogram is very poor at detecting small to moderate sized IHC lesions and that thresholds in quiet only begin to rise after the vast majority of IHC have been destroyed. Apparently, only a few IHC and type I neurons are needed to detect a tone in a quiet environment. The important implication of the above results is that DPOAE and pure tone audiograms, two of the most commonly used techniques for assessing hearing, are insensitive to profound IHC/type I neuron damage. This suggests additional measures are likely necessary to fully assess auditory function.

## IHC lesions have little effect on the cochlear microphonic

**Figure 4:**
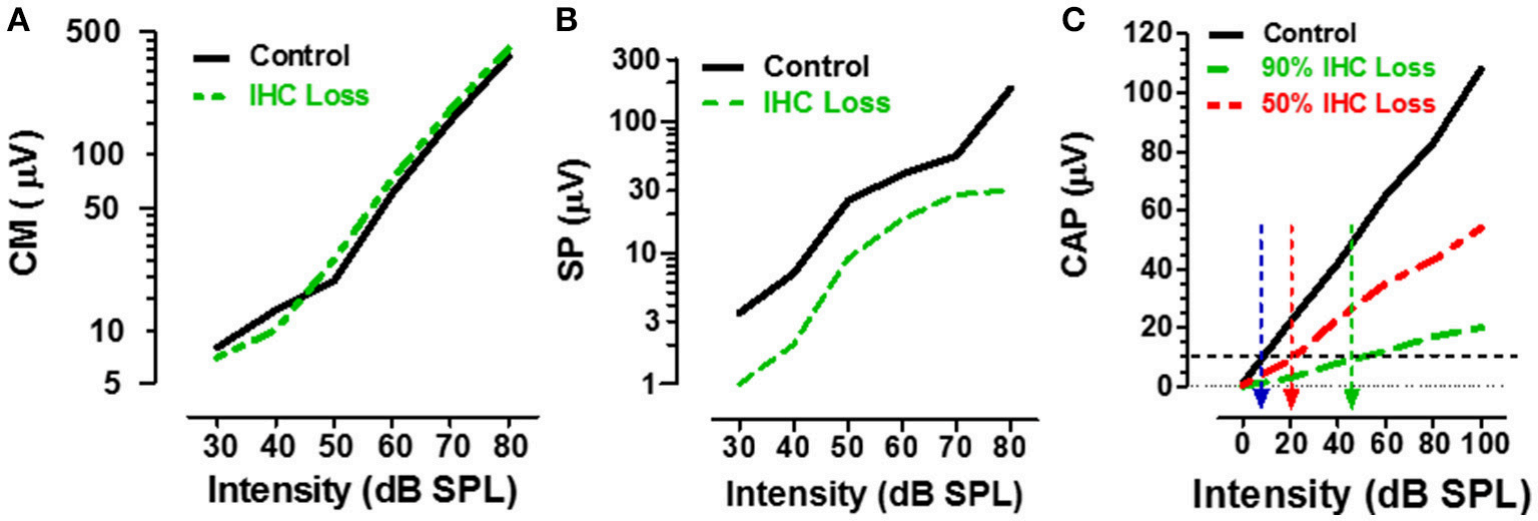
**(A)** Schematic showing the CM input/output functions in control (black) vs. carboplatin-treated (green) chinchillas with large IHC loss but intact OHC. IHC loss had little effect on CM amplitude. **(B)** Schematic illustrating the SP input/output functions in control (black) vs. carboplatin-treated (green) chinchillas with large IHC lesion with retention of OHC. IHC lesion caused a large reduction (~60%) in SP amplitude. **(C)** Schematic showing the CAP input/output function in control (black) and carboplatin-treated chinchillas with a large (~90%, green) or moderate (~50%, red) IHC lesion and intact OHC. IHC loss resulted in a large decrease in CAP amplitude; the amplitude reduction was proportional to IHC loss. Black horizontal dashed line at 10 μV used to derive CAP threshold for control group (~10 dB SPL, blue arrow) vs. groups with 50% IHC loss (~20 dB SPL, red arrow) or 90% IHC loss (~45 dB SPL, green arrow). Data schematized from Trautwein et al. (1996), Wang et al. (1997), and Durrant et al. (1998).

ECochG recorded from the ear canal, round window or within the cochlea, provides researchers and clinicians with a powerful tool to assess the functional integrity of the sensory and neural structures in the cochlea. The cochlear microphonic (CM), an AC receptor potential that mirrors the waveform of the acoustic stimulus, is predominantly generated by the OHC with only a small contribution from IHC (Dallos et al., 1972). Given that carboplatin preferentially damages the IHC and does not alter DPOAE, one would predict that the CM amplitude would be largely unaffected by carboplatin treatments that primarily target the IHC. Indeed, when the CM was recorded from the round window of carboplatin treated chinchillas with large IHC lesions but near complete retention of OHC, CM input/output functions were nearly identical to control as schematized in Figure 4A (Trautwein et al., 1996; Wang et al., 1997). These results indicate that the IHC contribute little to the generation of the CM and that the CM cannot be used to assess IHC function.

## IHC lesion suppresses the summating potential

The summating potential (SP), reflected as a sound-evoked DC shift near stimulus onset, is thought to be generated predominantly by the IHC receptor potential with a much smaller contribution from OHC (Russell and Sellick, 1983; Zheng et al., 1997). Given that carboplatin preferentially damages the IHC, one would predict that SP amplitude would be greatly reduced in animals with large carboplatin-induced IHC lesions. To test this hypothesis, the SP evoked by tone bursts was recorded from the round window of carboplatin treated chinchillas. In animals with large IHC lesions and complete retention of OHC, SP amplitude was greatly reduced (~60%) compared to controls as schematized by the SP input/output function in Figure 4B (Durrant et al., 1998). Destruction of both IHC plus OHC resulted in a further decline in SP amplitude. These results provide further confirmation that the SP is generated presynaptically primarily by IHC and this component of the ECochG can be used to assess the functional status of IHC.

## IHC loss depresses the compound action potential

The auditory nerve compound action potential (CAP), consisting of two negative peaks (N1 and N2), is the most widely studied component of ECochG. The CAP is most effectively elicited by acoustic stimuli with rapid rise time and is thought to reflect the synchronized onset response of type I auditory nerve fibers (Dallos, 1973; Zheng et al., 1996). Since the amplitude of the CAP is a postsynaptic response that depends on the release of excitatory neurotransmitter from the IHC, damage to the IHC would be predicted to greatly reduce the CAP. To test this hypothesis, CAP input/output functions were recorded from carboplatin-treated chinchillas with different degrees of IHC damage (Trautwein et al., 1996; Wang et al., 1997). In cases where most IHC were destroyed (80–90%) and most OHC were present, the amplitude of the CAP was greatly reduced compared to controls, whereas moderate (~50%) IHC loss resulted in a modest amplitude reduction as schematized by the CAP input/output functions in Figure 4C. These results indicate that the reduction in CAP amplitude is proportional to the degree of IHC loss (Wang et al., 1997; Qiu et al., 2000). CAP thresholds can be derived from the input/output functions using an amplitude criterion of 10 μV. In the schematic (Figure 4C), CAP threshold was ~10 dB SPL in the control group (blue arrow) and ~20 dB SPL in the carboplatin group with 50% IHC (red arrow). These results suggest that auditory nerve fiber thresholds are only slightly increased despite the moderate to severe IHC lesion. However, in cases where ~90% of the IHC were missing and very few nerve fibers would be available to generate a synchronized CAP response, the CAP threshold had increased to ~45 dB SPL (green arrow). These results suggest that the CAP amplitude and CAP threshold have the greatest utility for detecting damage to the IHC/type I auditory nerve fibers.

## Acoustically responsive auditory nerve fibers have low thresholds and are sharply tuned

**Figure 5:**
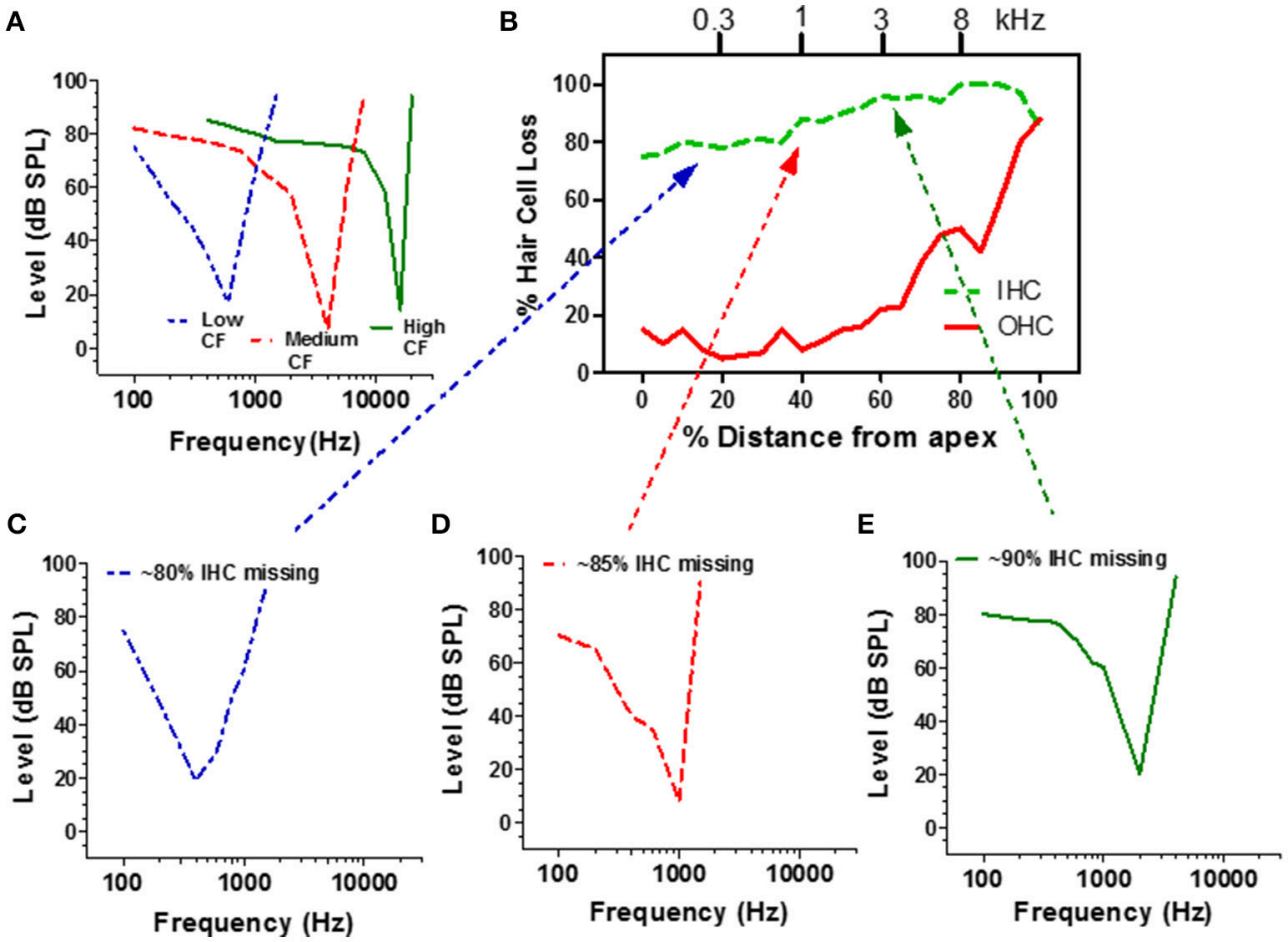
**(A)** Schematic of a low, medium and high CF auditory nerve fiber frequency-threshold tuning curves. The neuron's CF maps to the tonotopic map of the cochlea (frequency-place map upper x-axis, **B**). **(B)** Schematic cochleogram showing the percentage of missing IHC and OHC in a chinchilla treated with a high dose of carboplatin that destroyed approximately 80–98% of the IHC; damage was more severe in the basal half of the cochlea. Carboplatin also damaged OHC in the base (60–100%). **(C–E)** Schematic of auditory nerve fiber tuning curves from carboplatin treated animals with CFs near 300 Hz (**C**, blue), 1000 Hz (**D**, red), and 3000 Hz (**E**, green). Dotted lines relate each neuron's CF to IHC damage on tonotopic map (upper x-axis, **B**). Data schematized from Wang et al. (1997).

High impedance microelectrodes can be used to record the all or none spike discharges from single auditory nerve fibers as they leave the cochlea and enter the cochlear nucleus. Since each type I auditory nerve fiber contacts a single IHC, the neural output of a fiber reflects the activity from a discrete region of the basilar membrane. When tone bursts are used to measure the response of a single auditory nerve fiber, one can map out the frequency-intensity combinations that are just capable of evoking a response, the so-called frequency-threshold tuning curve (Salvi et al., 1982, 1983; Wang et al., 1997). Each tuning curve is characterized by a low threshold, narrowly tuned tip (Figure 5A). The frequency with the lowest threshold at the tip is the characteristic frequency (CF). The tuning curves of high CF and medium CF neurons are characterized by a steep high frequency slope above CF. Thresholds below CF also rise steeply, but gradually give rise to a high-threshold, broadly tuned tail. The tuning curves of low-CF neurons are more symmetrical and lack the broad low-frequency tail (Wang et al., 1997; Salvi et al., 1983, 1982).

Extensive damage to the IHC could conceivably affect the mechanical properties of the basilar membrane and alter the tuning and sensitivity of auditory nerve fibers. To evaluate this possibility, recordings were made from carboplatin-treated chinchillas with extensive IHC loss along the entire length of the cochlea as well as some OHC loss in the base of the cochlea as schematized in Figure 5B (Wang et al., 1997). When a microelectrode was advanced through the auditory nerve bundle, comparatively few acoustically responsive nerve fibers were encountered during the penetration, presumably due to the extensive loss of IHC and type I nerve fibers. However, when an acoustically responsive nerve fiber was encountered, CF-thresholds were low and tuning curve shapes were similar to those from normal control ears (Figures 5C–E; Wang et al., 1997). Thus, despite the massive IHC loss, the remaining IHC and type I neurons had low thresholds and sharp tuning which may explain why behavioral thresholds were relatively normal notwithstanding the large IHC loss. Apparently, sounds can be detected in quiet with only a weak signal from the few remaining IHC and type I neurons. Despite normal thresholds and tuning, spontaneous and suprathreshold responses from intact auditory nerve fibers were decreased in carboplatin-treated animals, indicative of subtle damage to surviving IHCs, and/or type I neurons (Wang et al., 1997).

## Central gain compensates for auditory deprivation

**Figure 6:**
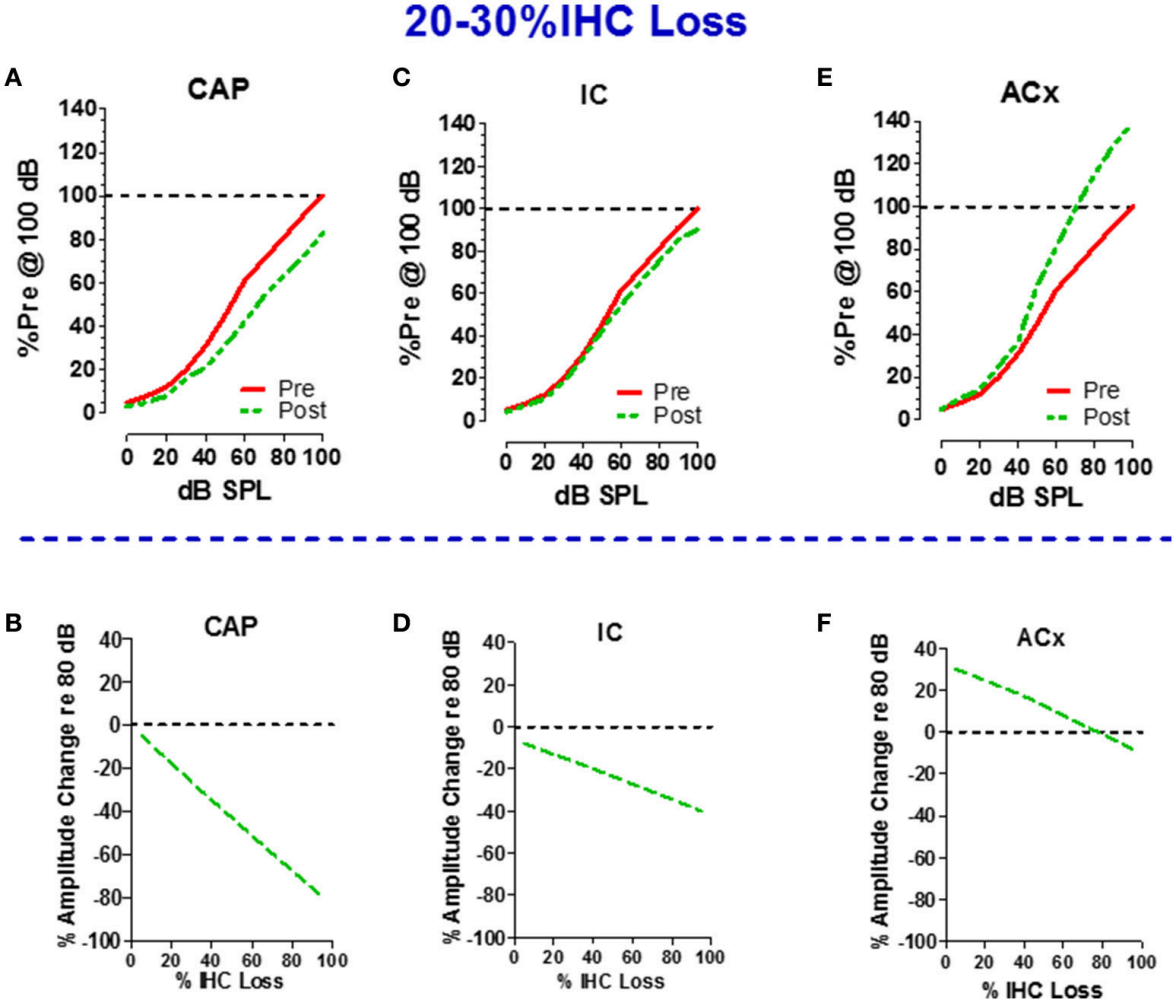
**Schematics in upper half illustrate the input/output functions recorded from the (A)** round window of the cochlea (compound action potential, CAP), **(C)** inferior colliculus (IC) and **(E)** auditory cortex (ACx) before and after carboplatin treatment that induced 20–30% IHC loss. Amplitude of local field potentials (LFPs) expressed as a percentage of the pre-treatment amplitude measured at 100 dB SPL. All pre-treatment amplitudes equal 100% at 100 dB before carboplatin treatment. Schematics in lower half show the percent change in amplitude of the LFPs recorded at 80 dB SPL vs. percent IHC loss; plots show result for the cochlear CAP **(B)**, IC **(D)**, and ACx **(F)**. Values above the dashed horizontal line in panel F indicate that at 80 dB SPL LFPs in the ACx were larger than normal for small to moderate size IHC lesion, but response were smaller than normal for IHC lesions >80%. Negative slopes indicate that LFP measured at 80 dB SPL decrease as IHC lesions increase. The decrease in amplitude was greatest for the CAP and least for the ACx. Data schematized from Qiu (1998) and Qiu et al. (2000).

If 75% of the IHC and type I neurons were destroyed, the central auditory pathway would receive only 25% of its normal input, a condition that would lead to a severe case of auditory sensory deprivation. A shout to a carboplatin-deafferented ear would likely be perceived as muffled unless there was some form of compensation to boost the weak neural signal. To determine how the central auditory system deals with diminished neural input from a carboplatin-damaged cochlea, recordings were made from chronically implanted electrodes in the cochlea (CAP), inferior colliculus (IC), and auditory cortex (ACx) of awake chinchillas before and after carboplatin treatment (Qiu et al., 2000). The schematics in the upper half of Figure 6 show the local field potential (LFP) input/output functions for the CAP (panel A), IC (panel C) and ACx (panel E) pre- and 5 weeks post-carboplatin treatment. The results portrayed in the upper half are representative data obtained from animals with mild IHC lesions of 20–30%. To facilitate a comparison across animals and conditions, the amplitudes are expressed as a percentage of the pre-treatment amplitude at 100 dB SPL. Consequently, all the pre-treatment values equal 100% at 100 dB SPL. In cases where 20–30% of the IHC were destroyed, the CAP amplitudes were smaller than normal. At 100 dB, the post-carboplatin CAP was reduced ~20% (80% of normal). Figure 6B is a schematic that shows the percent change in CAP amplitude at 80 dB as a function of percent IHC loss. CAP amplitude declines rapidly with IHC loss and the response is almost completely abolished with a loss of 90%. If the output of the auditory nerve was simply relayed up the central auditory pathway, the responses in the IC and ACx would mirror the CAP. Inspection of responses from the IC shows that the post-carboplatin input/output function is only slightly below the pre-treatment curve (Figure 6C; Qiu et al., 2000). The schematic in Figure 6D shows the percent change in IC amplitude at 80 dB vs. percent IHC loss. The slope of the IC function is roughly half that of the CAP, i.e., IC amplitude at 80 dB was only reduced ~40% compared to ~80% for the CAP. Carboplatin produced the most striking changes in the ACx where the post-exposure amplitudes were larger than pre-treatment values as schematized in Figure 6E. This cortical hyperactivity was dynamic, developing gradually over several days to weeks (Qiu et al., 2000). When the percent change in ACx amplitude at 80 dB is compared to percent IHC loss (Figure 6F), post-carboplatin amplitudes were 20–30% larger than normal (enhanced) with small to moderate IHC lesions and remarkably, only slightly below normal with near complete IHC lesions.

Taken together, these results indicate that the signal from the cochlea is progressively amplified as it is relayed to the central auditory pathway eventually leading to hyperactivity in the ACx. These findings are consistent with recent reports showing central gain enhancement with various forms of cochlear pathology; however, an unusual feature of carboplatin is that hearing thresholds are largely unaffected by the cochlea pathology (Salvi et al., 1990, 2000b; Sun et al., 2009; Stolzberg et al., 2011; Yuan et al., 2014; Brotherton et al., 2015; Chen et al., 2016). Interestingly, similar perceptual and electrophysiological changes were observed in a recent study examining ouabain treatment in mice, which selectively destroys type-1 SGN (Chambers et al., 2016). Animals with a unilateral lesion of >95% of afferent nerve fibers maintained relatively normal sound detection, likely due to a progressive recovery of sound-evoked activity along the central auditory pathway. Like the above results with carboplatin treatment (Figure 6), ouabain treatment greatly diminished auditory nerve responses while neural response were partially recovered in the IC and almost completely recovered, and in some cases enhanced, at the level of the ACx. Thus, cochlear damage appears to trigger a cascade of neuroplastic changes in the central auditory pathway to compensate for the reduced neural output from a damaged cochlea. Increasing the amplitude of a weak signal would make it easier for the ACx to detect sounds; this may explain why mild to moderate IHC and/or SGN loss has so little effect on auditory thresholds.

## Decreased inhibition in the auditory cortex

**Figure 7:**
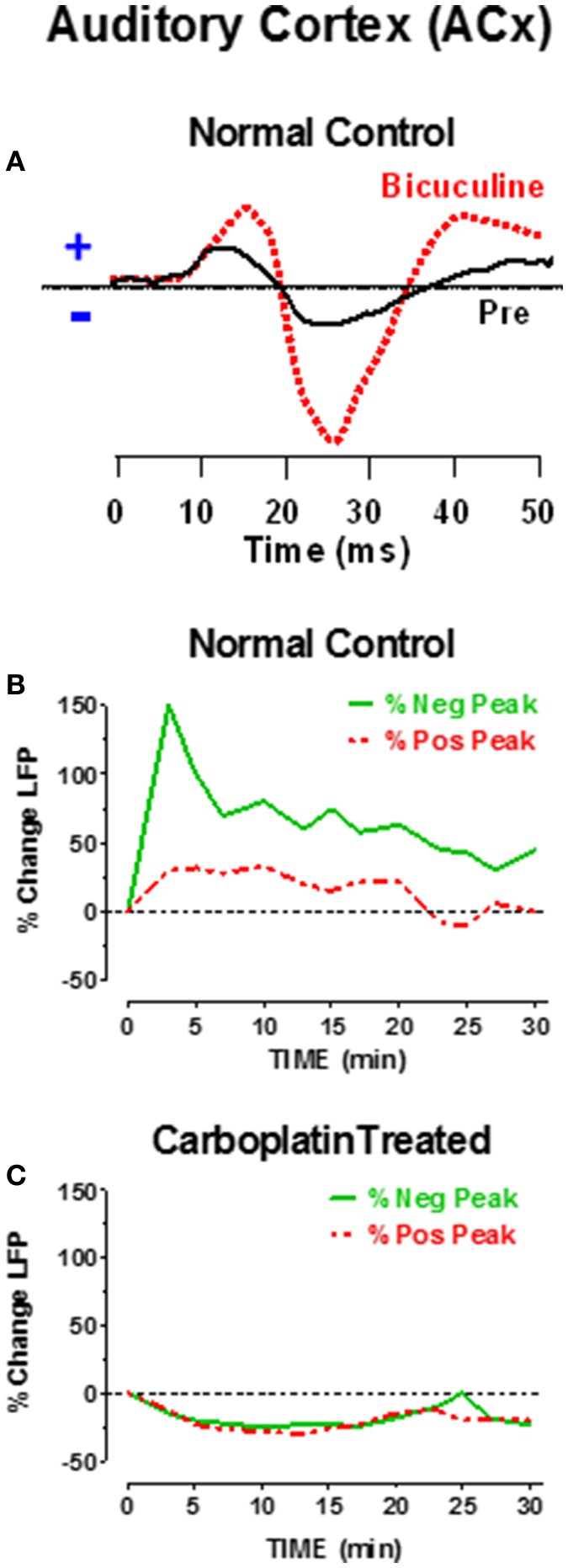
**(A)** Schematic of sound-evoked local field potentials (LFPs) from the auditory cortex (ACx) before (pre, solid black line) and after applying bicuculline (dashed red line) to the surface of the ACx of a normal control. Note increase in positive and negative peaks in the ACx waveform; increase in negative peak was larger than positive peak. **(B)** Percent change in positive and negative peaks in the LFP after applying bicuculline to the ACx. Bicuculline caused a large increase in positive and negative peaks. Largest increase occurred approximately 5 min post-treatment. Amplitudes gradually recovered with bicuculline washout. **(C)** Percent change in LFP after applying bicuculline to the ACx of chinchillas that had been treated with a moderate dose of carboplatin 1–2 months earlier. Bicuculline failed to induce an increase in cortical LFP, but instead induced a small decrease in the LFP which partially recovered 30 min after applying bicuculline. Data schematized from Salvi et al. (2000a, 2014).

Mechanistically, the heightened level of sound-evoked activity in the ACx of carboplatin-treated chinchillas could be due to increased excitation and/or decreased inhibition (Milbrandt et al., 2000; Suneja et al., 2000; Vale and Sanes, 2002; Sanes and Kotak, 2011). Gamma aminobutyric acid (GABA), a potent and ubiquitous inhibitory neurotransmitter, is heavily expressed in the central auditory system and ACx (Hendry and Jones, 1991; Prieto et al., 1994; Ling et al., 2005; Sacco et al., 2009) Neonatal sensorineural hearing loss reduces the number of GABAa receptors in the plasma membrane of layer 2/3 neurons in ACx (Sarro et al., 2008); this would decrease GABAa-mediated inhibition and may contribute to the hyperactivity seen in the ACx with cochlear hearing loss. To determine if the sound-evoked hyperexcitability in ACx was due to reduced GABAa-mediated inhibition, we measured LFPs in the ACx of normal and carboplatin-treated chinchillas while manipulating inhibitory tone (Salvi et al., 2000a, 2014). When bicuculline, a potent GABAa antagonist was applied locally to the ACx, it increased the firing rate, broadened the tuning and lowered the threshold of many ACx neurons (Wang et al., 2000). Bicuculline applied to the surface of the ACx of normal-hearing chinchillas also dramatically increased the amplitude of the sound-evoked LFP in the ACx as schematized in Figure 7A; the amplitude enhancement was much greater for the negative peak than the positive peak. Figure 7B is a schematic that shows the time course and percent increase in the magnitude of the positive and negative peaks of the LFP response after bicuculline was applied to the ACx of a normal chinchilla. The maximum increase occurred ~5 min after bicuculline was applied to the ACx and the response gradually recovered toward baseline values over the following 30 min as bicuculline washed out. These results indicate that under normal conditions, GABA strongly inhibits sound-evoked responses in the ACx, but when GABAa receptors are blocked with bicuculline sound-evoked activity increases dramatically.

As noted above (Figure 6), sound-evoked responses in the ACx greatly increase after carboplatin treatment, potentially indicating that GABA mediated inhibition was already diminished. To investigate this possibility, bicuculline was applied to the ACx of chinchillas that had been treated with a moderate dose of carboplatin. As schematized in Figure 7C, bicuculline failed to increase the amplitude of the sound-evoked LFP in the ACx; instead there was a slight reduction that dissipated over time. Thus, carboplatin treatment appears to occlude the effects of bicuculline on ACx responses. Failure of bicuculline to increase sound-evoked activity could occur if there was a significant decline in GABAa receptors in the ACx, an interpretation consistent with previous findings in hearing impaired animals (Sarro et al., 2008). An alternative possibility is that less GABA is released from presynaptic neurons; however, this view is not supported by results from hearing impaired animals (Sarro et al., 2008). While altered inhibition has been observed in both cortical and subcortical auditory structures following noise-induced hearing loss (Milbrandt et al., 2000; Dong et al., 2010; Wang et al., 2011; Yang et al., 2011), it remains to be determined if changes to GABA-mediated inhibition are involved in the partial recovery of IC responses following carboplatin treatment.

## Critical band perceptual deficits with IHC loss

**Figure 8:**
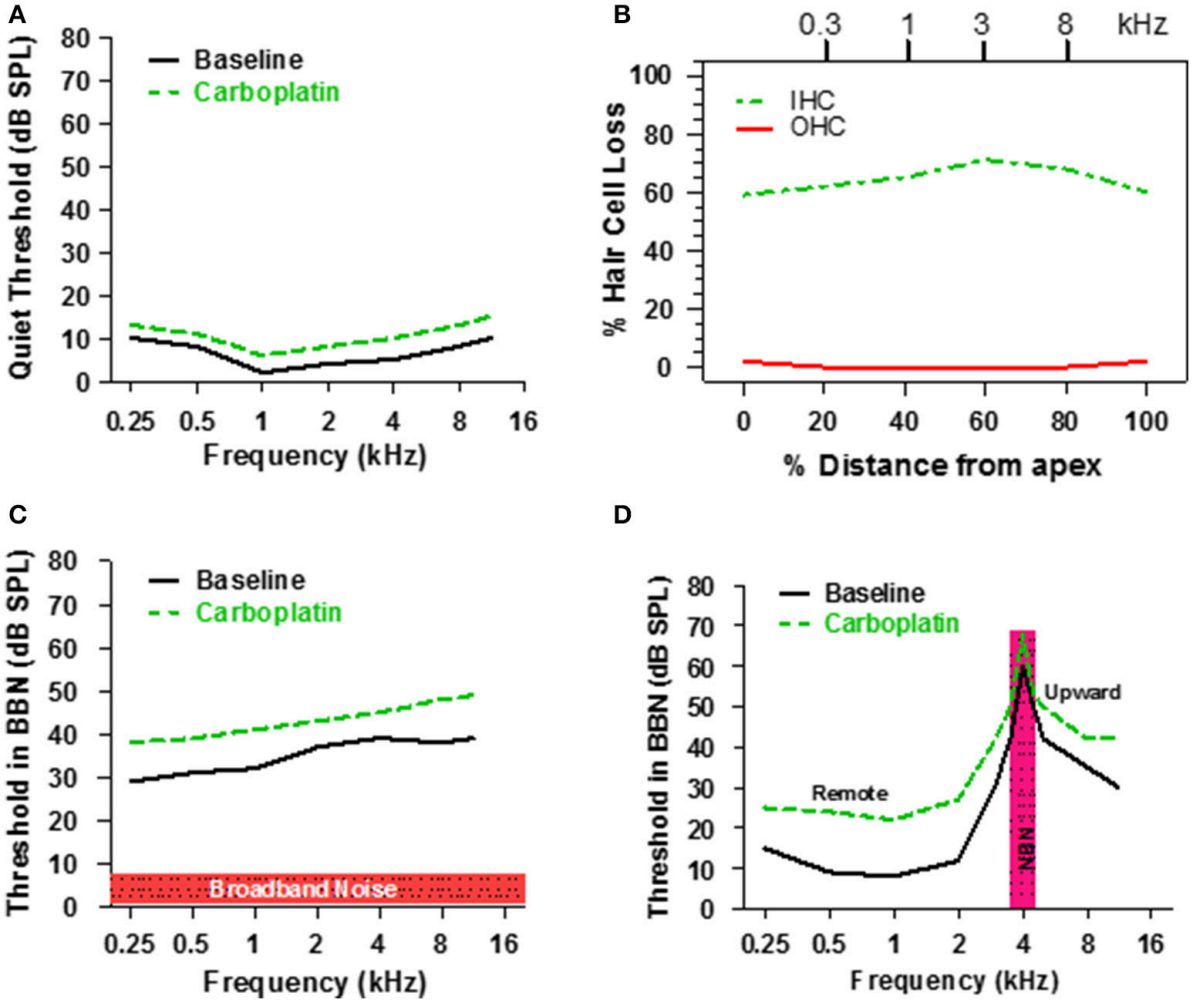
**(A)** Schematic illustrating threshold in quiet before and after a moderate dose of carboplatin. **(B)** Schematic representing the IHC and OHC loss after a moderate dose of carboplatin that induces a 60–70% IHC with little or no loss of OHC. **(C)** Schematic showing the threshold in 50 dB SPL broadband noise before and after moderate dose of carboplatin. **(D)** Schematic illustrating the thresholds measured in narrowband noise (100 Hz bandwidth) centered at 4 kHz before and after a moderate dose of carboplatin. Carboplatin-induced threshold elevations above 4 kHz reflect the upward spread of masking and those below 4 kHz reflect remote masking. Data schematized from Lobarinas et al. (2016).

Taken together, the above results suggest that only a fraction of IHC and type 1 nerve fibers are required for normal hearing thresholds in quiet (Figure 3) because activity from the few remaining intact nerve fibers, which maintain low thresholds and sharp tuning (Figure 5), is progressively amplified through the central auditory system (Figure 6). However, listening with few IHC and type I neurons might be extremely challenging in more difficult listening environments. Each IHC is contacted by 10–20 type I nerve fibers resulting in considerable redundancy in the information relayed by each IHC to the central auditory system. Moderate IHC and type I neural loss would greatly reduce this redundancy and information transfer to the auditory brain which could result in auditory processing deficits in conditions with decreased signal to noise ratio, such as detecting sounds in noisy environments. To test this hypothesis, chinchillas were trained to detect a tone burst in broadband noise (BBN), a technique often used to investigate the internal critical band filters (Scharf, 1961, 1970). Consistent with previous results, pure tone thresholds measured in quiet were only slightly higher (3–5 dB) than baseline after administering a dose of carboplatin that destroyed ~60–70% of the IHC as schematized in Figures 8A,B (Lobarinas et al., 2013, 2016). Pure tone thresholds were then measured in BBN with an overall SPL of 50 dB and a spectrum level of ~7 dB as schematized in Figure 8C. During baseline testing, tone thresholds in BBN increased with frequency up to around 8 kHz and then plateaued similar to previous results (Seaton and Trahiotis, 1975). After carboplatin treatment, tone thresholds in BBN increased at all frequencies; the 6–11 dB increase in signal to noise ratios was statistically significant (Lobarinas et al., 2016).

According to critical band theory, detection of the signal depends on the power in the signal relative to the power passing through the width of the critical band (Scharf, 1970). The carboplatin-induced increase in critical band values could result from a widening of the critical band. However, since sharp tuning is maintained at the auditory nerve (Figure 5) and IC (Wake et al., 1996b), the absence of band widening would not alter the amount of noise passing through the critical band and therefore not alter the signal to noise ratios. However, it is still possible that broader neural tuning could emerge at the level of the ACx due to loss of GABA-mediated inhibition (Wang et al., 2002). Alternatively, the increase in signal to noise ratios (Figure 8C) could result from an increase in central gain because the total amount of noise passing through a filter is the product of the bandwidth times the gain.

## Narrow band noise masking with IHC loss

Another approach used to assess the frequency selectivity of the auditory system is to measure tone burst thresholds at frequencies below, at and above the center frequency of a narrow band noise (NBN); a plot of threshold vs. frequency in the presence of the masker defines the NBN masking profile (Egan and Hake, 1950). The solid line in Figure 8D is a schematic showing a typical masking profile for a NBN (100 Hz bandwidth) centered at 4 kHz. The baseline NBN masking profile in a normal chinchilla is asymmetric. Masked thresholds are highest at the frequency of the masker, but decrease rapidly for frequencies below 4 kHz. In contrast, masked thresholds decline gradually at frequencies above the 4 kHz masker resulting in considerable upward spread of masking. To determine if the NBN masking profile was altered by the loss of IHC, chinchillas were treated with a moderate dose of carboplatin that destroyed 60–70% of the IHC (Figure 8B), which had little effect on thresholds in quiet (Figure 8A). Following carboplatin-treatment, threshold at the 4 kHz NBN masker increased approximately 10 dB, a result consistent with the BBN masking pattern. A 10–12 dB threshold increase also occurred at frequencies above the masker. Thus, the tip and high-frequency leg of the NBN masking pattern were shifted upward, but the bandwidth of the tip was essentially unchanged indicating that frequency selectivity was normal near 4 kHz. Paradoxically, masked thresholds were also elevated 10–15 dB at frequencies (0.25–2 kHz) far below the 4 kHz masker, a phenomenon known as remote masking. It has been suggested that remote masking arises from OHC electromotility and nonlinear motion of the basilar membrane because conditions that disrupt the OHC reduce remote masking (Cervellera and Quaranta, 1982; Salonna et al., 1992; Quaranta et al., 1999). Since OHC appeared functionally intact, our results suggest that IHC/type I neurons normally suppress remote masking as their loss results in greater remote masking. Thresholds in NBN were elevated over a broad range of frequencies, a pattern at odds with basilar membrane mechanics. An alternative explanation for the widespread increase in masked threshold is that it is due to the loss of GABA-mediated inhibition in the ACx since pharmacologic blockage of GABA-mediated inhibition results in broadening of ACx tuning curves both above and below CF (Wang et al., 2002).

## Synopsis

Much of the basic auditory research over the past century has focused on the anatomy and physiology of the cochlea. As a result, we now have noninvasive functional tests such as DPOAE to evaluate the status of the OHC in the cochlea. DPOAE are maintained at normal levels (Figure 2) if cochlear damage is confined to IHC, but rapidly decline if the OHC are also destroyed (Hofstetter et al., 1997a). The CM component of ECochG can also be used to evaluate the functional status of OHC as well. Selective destruction of the IHC with carboplatin had no measureable effect on the CM (Figure 4A), consistent with the notion that OHC are the dominant generators of this potential. In addition, the SP of ECochG provides a powerful tool for evaluating activity of IHC, since IHC loss results in a massive decline, but not complete abolition, of this potential (Figure 4B; Durrant et al., 1998). The CAP, the neural component of ECochG, is useful for assessing the sensitivity and global neural output of the cochlea. The decline in CAP amplitude is roughly proportional to IHC loss (Figure 4C) whereas changes in CAP threshold are more difficult to assess because a large decline in CAP amplitude can make it difficult to clearly identify the CAP.

One of the most remarkable psychoacoustic findings from the series of carboplatin studies reviewed here was that hearing thresholds in quiet were nearly normal despite the massive loss of IHC and type I neurons. Our auditory nerve fiber recording (Figure 5) and psychoacoustic (Figure 3) results suggest that only a few normal functioning IHC and type I neurons are needed to hear a tone in a quiet environment. How is it that we can hear so well when only a few IHC and type I neurons are connected to the brain? The answer to this question may relate to the fact that we perceive sounds not just with the cochlea, but also with our brain. The decrease in the neural output of the cochlea likely triggers a series of homeostatic processes at multiple stages of the auditory pathway that amplify these weak signals so that by the time it reaches the IC or ACx, sound-evoked responses are normal or even supra normal (Figure 6; Qiu et al., 2000; Jiang et al., 2016). The increases in gain seem to be most pronounced with mild to moderate IHC lesions, where the largest increase in ACx response occurred (Figures 6E,F). Since carboplatin-induced damage results in relatively matched bilateral lesions, it is possible that recovery from more severe IHC loss may be observed if lesions were restricted to one ear, as was found recently for a ouabain model of auditory neuropathy (Chambers et al., 2016). However, the fact that extensive recovery of sound encoding (and even over amplification as in the ACx) is observed in carboplatin-treated animals with bilateral lesions suggests that central gain enhancement is not limited to ear- or input-specific competitive changes but can also arise from a balanced loss of input to both ears. Our results suggest that, at least for carboplatin-induced cochlear damage, enhanced central gain and neural amplification is due in part to the loss of GABA-mediated inhibition in the ACx. However, a plethora of additional mechanisms operating at multiple levels of the auditory system are likely to be involved as well (Suneja et al., 2000; Chen et al., 2007; Peppi et al., 2012; Auerbach et al., 2014).

There is currently tremendous scientific and clinical interest in a form of hidden hearing loss termed synaptopathy that affects the synaptic ribbon at the base of the IHC, glutamatergic receptors located on type I auditory nerve terminals and neurotrophin3 which provides trophic support for SGN (Liberman et al., 2011; Kujawa and Liberman, 2015; Shaheen et al., 2015; Viana et al., 2015; Shi et al., 2016a,b; Suzuki et al., 2016). Identifying the unique perceptual deficits associated with this condition will provide additional tools for identifying individuals with normal clinical audiograms that nonetheless have significant auditory processing disruptions. Although the histopathologies associated with carboplatin damage in the chinchilla are likely somewhat different than those with pure synaptopathy, our psychophysical studies suggest that a simple tone in BBN noise detection task, something that can be accomplished with a clinical audiometer, may be a sensitive method for identifying damage confined to the IHC and/or SGN. Tests of remote masking might also be useful since remote masking increased in chinchillas with selective damage to IHC and type I neurons whereas remote masking decreases with age-related hearing loss and salicylate ototoxicity, conditions likely to involve OHC pathology.

Why does loss of IHC/type I neurons result in difficulties hearing in noise? While remaining auditory nerve fibers maintain normal tuning and thresholds following carboplatin treatment (Figure 5), there is evidence for reduced spontaneous and maximum driven firing rates, which could lead to coding deficits in noisy conditions (Wang et al., 1997). A recent study has demonstrated that auditory nerve fibers with low spontaneous firing rates are preferentially damaged by noise exposure that causes hidden hearing loss (Furman et al., 2013). These nerve fibers are characterized by a relatively large dynamic range and wide threshold distribution and are therefore well-equipped for coding sounds in noisy backgrounds, suggesting that selective loss of these nerve fibers could lead to problems hearing in noisy environments. Interestingly, carboplatin treatment results in a decrease in the median spontaneous firing rate of auditory nerve fibers, shifting the population in favor of lower spontaneous rates rather than higher (Wang et al., 1997). This suggests that the difficulties hearing in noise experienced by carboplatin-treated chinchillas is not likely due to the loss of a specific class of auditory neurons, contrary to what is seen with noise-induced hidden hearing loss, but may be due in part to the reduced maximum driven rates seen at high sound levels (Wang et al., 1997).

Adding to the complexity of cochlear hearing loss is the fact that the central auditory system attempts to compensate for peripheral change by turning up its gain. While central gain enhancement is able to restore normal hearing under quiet conditions (Figure 3), it may not adequately compensate for peripheral dysfunction in more difficult sound environments (Figure 7) or in response to temporally complex stimuli (Lobarinas, 2006; Chambers et al., 2016). This could be because central gain enhancement is most prominent in higher auditory areas that lack the temporal precision required to follow rapid acoustic fluctuations that brainstem and peripheral auditory centers are optimized for. Alternatively, it could be a byproduct of the mechanisms by which gain enhancement is achieved. For instance, while a loss of cortical inhibition may allow for recovery of rate-intensity coding following hearing loss, it could also result in temporal coding deficits that may contribute to impaired speech perception and difficulties hearing in noisy conditions (Wehr and Zador, 2003; Scholl and Wehr, 2008).

Central adaptation to hearing loss is also likely crucial in the development of auditory perceptual disorders like tinnitus and hyperacusis. From a clinical perspective, it would be difficult to account for loudness recruitment or hyperacusis (loudness intolerance) based on the neural responses seen in the damaged cochlea (Figures 1A,B). The large gain enhancements seen in the ACx seem particularly relevant to loudness hyperacusis. To our knowledge, no one has tested for evidence of hyperacusis in carbolpatin-treated chinchillas to determine if loudness intolerance is related to carboplatin-induced hyperactivity. However, we have found a striking correlation between salicylate-induced hyperactivity in the central auditory system of rats with behavioral evidence of loudness hyperacusis (Chen et al., 2014, 2015). While enhanced central gain can compensate for the reduced neural output of the cochlea, too much gain at low sound levels could contribute to tinnitus whereas excess gain at high levels may give rise to loudness hyperacusis.

## Author contributions

WS, DD, GC, EL, JW, KR, and BA finished the experiment. RS, WS, DD, GC, EL, and KR analyzed the results. RS, BA, and WS wrote and edited the paper.

### Conflict of interest statement

The authors declare that the research was conducted in the absence of any commercial or financial relationships that could be construed as a potential conflict of interest.
